# Pro-Inflammatory Activation of a New Immortalized Human Microglia Cell Line

**DOI:** 10.3390/brainsci9050111

**Published:** 2019-05-15

**Authors:** Marta Chiavari, Gabriella Maria Pia Ciotti, Pierluigi Navarra, Lucia Lisi

**Affiliations:** 1 Institute of Pharmacology, Catholic University Medical School, L.go F Vito 1, 00168 Rome, Italy; m.chiavari@hotmail.it (M.C.); gabriella.ciotti@hotmail.it (G.M.P.C.); lucia.lisi@unicatt.it (L.L.); 2 Fondazione Policlinico Universitario Agostino Gemelli, L.go F. Vito 1, 00168 Rome, Italy

**Keywords:** human microglia, cytokines, M1 phenotype, mTOR

## Abstract

The characterization of human microglia has been hampered by poor availability of human cell sources. However, microglia is involved in the physiopathology of multiple sclerosis, Alzheimer’s disease, Parkinson’s disease, HIV dementia, retinal degenerative diseases, cancer, and many other conditions. Therefore, there is an important need to have experimental paradigms of human microglia characterized and usable to study the role of microglia in the different pathologies in which it is involved. In the present work, we carried out an extensive characterization of Immortalized Human Microglia—SV40 cell line (IMhu), marketed by Applied Biological Material. The functional response of IMhu to a large variety of stimuli was studied. In particular, we investigated morphology, mortality, and changes in the production of different cytokines and chemokines, both under basal conditions and after stimulation. Moreover, western blotting analysis was conducted on phospho-mTOR (Ser 2448) and downstream parameters, p-P70S6K and 4EBP1, in order to understand if IMhu can be used for evaluations of mTOR pathway. In conclusion, IMhu cells proved to be a useful experimental model to investigate the physiopathology of inflammatory disease that involved microglia cells, including pathological conditions that involved the mTOR pathway.

## 1. Introduction

Microglia, myeloid cells resident in the brain parenchyma, represent the first line of immune defense within the central nervous system (CNS), playing a role in synaptic architecture and neurogenesis [1,2]. These cells are often described as either resting (i.e., ramified) or activated, but these terms fail to convey the dynamic remodeling of their fine processes and constitutive immune-surveillance activity. Chronic activation of microglia is believed to exert detrimental effects, which underlie the pathogenic mechanisms common to many neurodegenerative diseases. Microglia is involved in the physiopathology of multiple sclerosis, Alzheimer’s disease, Parkinson’s disease, HIV dementia, retinal degenerative diseases, cancer, and many other conditions [3,4,5].

At present, different phenotypes of microglia are described, each having specific functions. In particular, microglia is classified into four main states or phenotypes. Under certain pathological conditions, microglial cells are polarized into the M1 type, releasing pro-inflammatory factors and causing neuro-inflammatory responses. When inflammation fades away, or in the presence of a tumor microenvironment, microglia shift into the alternatively activated M2 phenotype, which in turn can be distinguished into the M2a, M2b, or M2c subtypes. Overall, the M2 phenotype is associated to neuroprotection and tumor growth [6,7].

Microglial activation and function were extensively investigated in animal experimental models (both rats and mice). In contrast, the characterization of human microglia has been hampered by poor availability of human cell sources. To overcome this problem, in the last few years human immortalized microglial cell lines have been developed, and different human microglia cell lines have been commercialized.

The Immortalized Human Microglia—SV40 cell line (IMhu) is marketed by Applied Biological Material (ABM). IMhu are derived from Primary Human Microglia Cells (>99% purity), and maintained the microglial marker CD68 and NGF, as shown by RT-PCR (https://www.abmgood.com/Immortalized-Microglia-SV40-T0251.html#documents). These cells are suitable for studying the role of human microglia in health and disease. Thus far, iMhu have been described as an experimental paradigm in a number of reports [5,8,9,10,11,12,13,14,15].

In the present work, an extensive characterization of iMhu has been carried out; the response of these cells to pro-inflammatory and anti-inflammatory stimuli has been evaluated. Many conditions of M1 and M2 activation have been tested. Moreover, 2-h experiments in the presence of M1 stimuli were specifically addressed to see whether iMhu cells could be used for evaluation of the pro-inflammatory activated mTOR pathway.

## 2. Methods

### 2.1. Materials

Cell culture reagents (Dulbecco’s modified Eagle’s medium (DMEM—Cat. No.: 10-013-CVR), DMEM-F12 (Cat. No.: D6421 and fetal calf serum (FCS—Cat. No.: 10091-148)) were from Invitrogen Corporation (Paisley, Scotland) while Prigrow III (Cat. No.: TM003) was from Applied Biological Materials Inc. (Richmond, QC, Canada). Antibiotics were from Biochrom AG (Cat. No.: A2212—Berlin, Germany). The human recombinant interleukin 1β (IL1β—Cat. No 201-LB), human IFN γ, and recombinant human tumor necrosis factorα (TNFα—Cat. No MAB210-SP) were purchased from R & D System (Minneapolis, MN, USA). The human recombinant interleukin 4 (IL4—Cat. No.: ENZ-PRT180-0020) and human recombinant interleukin 13 (IL13—Cat. No.: ENZ-PRT183-0010) were purchased from Enzo Life Sciences, Inc. (Farmingdale, NY, USA). β-actin (clone AC-74—Cat. No.: A5316) mouse monoclonal antibody was from Sigma-Aldrich (St.Louis, MO, USA), rabbit polyclonal anti-phospho (ser-2448) mTOR (Cat. No.: NB600-607) was purchased from Novus Biological (Littleton, CO, USA), mouse monoclonal p-p70 S6K (Cat. No.: SC-8416) was purchased from Santa Cruz Biotechnology, Inc. (Santa Cruz, CA, USA), rabbit polyclonal 4EBP1 (Cat. No.: A300-501A) was purchased from Bethyl Laboratories, Inc (Montgomery, TX, USA), anti-rabbit was purchased from Jackson ImmunoResearch Laboratories, Inc. (Cat. No.: 111-035-045, West Grove, PA, USA), and anti-mouse was purchased from Sigma-Aldrich (Cat. No.: A3682—St.Louis, MO, USA).

### 2.2. Cell Cultures

Immortalized Human Microglia—SV40 (IMhu; Cat.No.: T0251) was purchased from Applied Biological Materials Inc (Richmond, QC, Canada). IMhu was grown in Prigrow III media containing 10% FCS and antibiotics in Applied Biological Materials Inc. (Richmond, QC, Canada) PriCoat™ T25 flasks (Cat. No.: G299) and seeded at 40,000 cell per cm^2^ density; experimental conditions were reached with DMEM-F12 at a low concentration of FCS (1%) in handmade Corning T25 coated flask with collagen I at 0.1mg/mL (Cat. No.: A10483-01—Gibco Life Technologies Corporation, Grand Island, NY, USA) concentration. Briefly, flasks were treated with collagen 0.1mg/mL in PBS with calcium and magnesium (Cat. No.: D1283—Sigma-Aldrich, St.Louis, MO, USA) for 1 h at 37 °C; after that, two washes in PBS with calcium and magnesium were needed and flasks were used to seed the cells. Cells were splitted at the 80% of the confluence. All the experiments received institutional approval.

### 2.3. M1 and M2 Stimulation

A mix of pro-inflammatory cytokines, namely 10 ng/mL TNFα, 50 ng/mL IL1β, and 20 ng/mL IFNϒ, referred to as TII group was used to reach a M1 stimulation. In particular, 24 h after the seeding IMhu cells were treated with TII and after another 24 h, the experiments were stopped.

Similarly, to reach M2 stimulation with IL13 in the range of concentrations 50–100–200 ng/mL, or with IL4 in the range of concentrations 10–20–40 ng/mL, the IMhu cells were activated 24 h after the seeding and the experiments were conducted for another 24 h.

All the experiments were carried out in Pigrow III, DMEM/F12 or in DMEM with 1% FBS.

### 2.4. Viability and Mortality

The cell viability was detected using RealTime-Glo™ MT Cell Viability Assay (Cat. No.: G9713—Promega, Madison, WI, USA) and the number of nonviable cells was detected using CellTox™ Green Cytotoxicity Assay (Cat. No.: G8742—Promega, Madison, WI, USA). The assays were conducted following the manufacturer’s instructions.

### 2.5. LDH Assay

At the end of selected experiments, cell viability was assessed by measurement of the activity of lactate dehydrogenase (LDH) released in the incubation media, using the CytoTox-96 kit from Promega (Cat. No.: G1780, Madison, WI, USA), according to the manufacturer’s instructions.

### 2.6. Urea Assay

Urea levels in IMhu cells were detected by the QuantiChrom Urea Assay kit (Cat. No.: DIUR-100—BioAssay System, Hayward, CA, USA), used according to the manufacturer’s instructions. Briefly after 24 h of incubation with the tested substances, an aliquot of cell culture media (50 µL) was mixed with 200 µL Urea Reagent (BioAssay system, Hayward, CA, USA) and the absorbance measured at 430 nm in a spectrophotometric microplate reader (PerkinElmer Inc., Waltham, MA, USA). A standard curve was generated during each assay in the range of concentrations 0–100 µg/mL using urea as standard. In this range, standard detection resulted linear, and the minimum detectable concentration of urea was 3.12 µg/mL. The protein content in each sample was determined by Bradford’s method (Cat. No.: 5000006—Bio-Rad, Hercules, CA, USA) using bovine serum albumin (BSA—Cat. No.: A2153—Sigma-Aldrich, St.Louis, MO, USA) as standard.

#### Multiple Cytokines Analysis

Levels of cytokines, chemokines, and growth factors from cell lysates were detected using the Proteome Profiler Human Cytokines XL kit (Cat. No.: ARY022B—R & D System, Minneapolis, MN, USA). The assay was conducted following the manufacturer’s instructions.

### 2.7. Western Immunoblot

The cells were lysed in RIPA buffer [1 mM EDTA (Cat. No.: E7889), 150 mM NaCl (Cat. No.: S9888, 1% igepal (Cat. No.: I3021), 0.5% sodium deoxycholate (Cat. No.: D-6750), 50 mM Tris–HCl, pH 8.0 (Cat. No.: T-3038) (Sigma-Aldrich, St.Louis, MO, USA), and 0.1% sodium dodecyl sulfate, SDS, (Cat. No.: 1610416—Bio-Rad, Hercules, CA, USA)] containing protease inhibitor cocktail diluted 1:250 (Cat. No.: P8340—Sigma–Aldrich, St.Louis, MO, USA). The protein content in each sample was determined by Bradford’s method (Bio-Rad, Hercules, CA, USA) using bovine serum albumin as standard. A 100 µg aliquot of protein was mixed with 4× Bolt™ LDS Sample Buffer (Cat. No.: B0007—Novex, Carlsbad, CA, USA) and 10× Bolt™ Sample Reducing Agent (Cat. No.: B0009—Novex, Carlsbad, CA, USA), boiled for 5 min, and separated through 7% polyacrylamide SDS gels. Apparent molecular weights were estimated by comparison to colored molecular weight markers (Cat. No.: LC5925—Invitrogen, Carlsbad, CA, USA). After electrophoresis, proteins were transferred to nitrocellulose membranes by iBlot™ 2 Gel Transfer Device (Invitrogen, Carlsbad, CA, USA). The membranes were blocked with 10% (*w*/*v*) low-fat milk (Cat. No.: 170-6404—Bio-Rad) in TBST (TBS Cat. No.: 28358—Thermo Scientific™ (Rockford, IL, USA); Tween 20 Cat. No.: P5927—Sigma-Aldrich, St.Louis, MO, USA) for 1 h at room temperature and incubated in the presence of the primary antibody overnight with gentle shaking at 4 °C for phosphorylated m-TOR and p-P70S6K while membranes were incubated in the iBind Flex Western Device (Cat. No.: SLF2000—Invitrogen, Carlsbad, CA, USA) for 4EBP1 and β-actin. Primary antibodies for phosphorylated-mTOR, 4EBP1, p-P70S6K, and β-actin were used at the final concentration of 1:1000. After the overnight incubation, primary antibody of p-m-TOR and p-P70S6K was removed, membranes washed 3 times in TBST, and further incubated for 1 h at room temperature in the presence of specific secondary antibody diluted 1:8000 for anti-mouse and 1:10,000 for anti-rabbit. Following three washes in TBST, bands were visualized incubating the membranes in ECL reagents (Cat. No.: 34580; Cat. No.: 32106—Thermo Scientific™, Rockford, IL, USA) and exposed to CL-XPosure™ Film (Cat. No.: 34090—Thermo Scientific™, Rockford, IL, USA). The same membranes were washed 3 times in TBST and used for 4EBP1 and β-actin immunoblot in the iBind™ Flex Western Device with primary antibody used at a final concentration of 1:1000 and secondary antibody diluted 1:3000 for anti-mouse and 1:15,000 for anti-rabbit.

### 2.8. Immunostaining

Cover glasses of 13 mm of diameter were coated with Collagen I 0.1 mg/mL or Poli-L-lysine 50 μg/mL (Cat. No.: A-003-E—Merck Millipore, Burlington, MA, USA) for 1 h at 37 °C, after that three washes in PBS with calcium and magnesium were needed and used to seed the IMhu at 50,000–100,000 cells per well concentration. After 24 h from the treatment, cells were blocked with PAF at 4% concentration in PBS with calcium and magnesium for 20 min at room temperature. After three washes in PBS with calcium and magnesium, cells were blocked with BSA and incubated in the presence of primary antibody. The incubation time was of 2 h for Phalloidin 1:400 (Cat. No.: P1951—SigmaAldrich, St.Louis, MO, USA) and overnight for CD11b (Cat. No: NB110-89474—Novus Biological, Littleton, CO, USA) 1:200; after three washes in PBS with calcium and magnesium in gentle shaking, cells were incubated with secondary antibody for 1 h and mounted with Vectashield with DAPI (Cat. No: H-1200—Vector Laboratories, Burlingame, CA, USA).

#### Statistical Analyses

Statistical analysis of the differences between pairs of groups was performed by Student’s t-test. For multiple comparisons, ANOVA analysis, followed by Bonferroni’s post-test, was used. Statistical significance was determined at α = 0.05 level. Differences were considered statistically significant when *p* < 0.05. All the experiments were repeated at least three times.

## 3. Results

In the present work, Immortalized Human Microglia—SV40 (IMhu) cell line—has been characterized under basal conditions and after cytokine stimulation.

**Figure 1:**
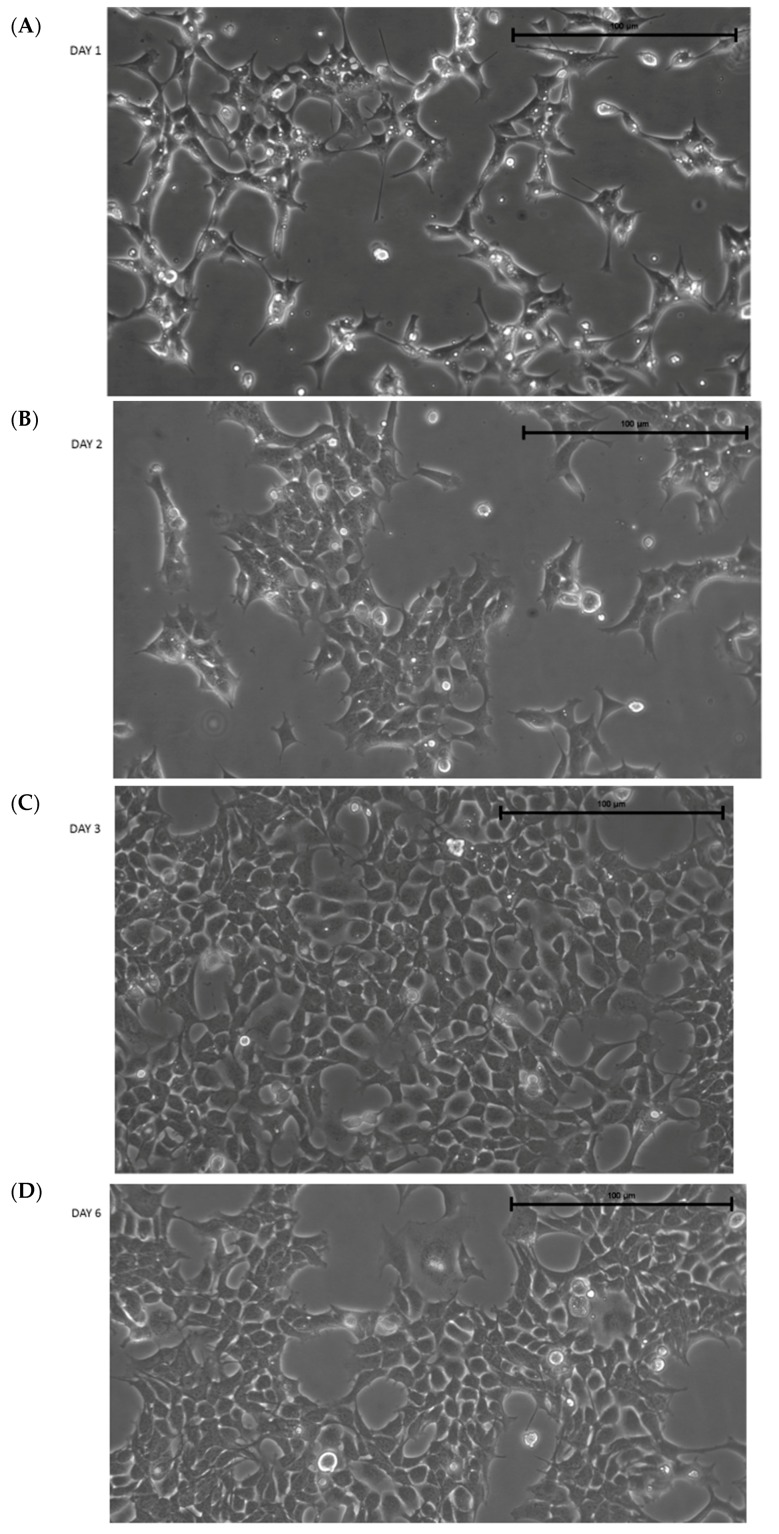
Morphology of IMhu. (**A**–**D**) Morphology of IMhu cells, observed by phase-contrast microscopy on days 1, 2, 3, and 6 after thawing. Magnitude 20×.

After thawing, IMhu cells present with a triangular shape, typical of microglia cells in a resting state. Figure 1 shows the morphology of cells 1, 2, 3, and 6 days after thawing. Figure shows that the cells reach confluence (by about 80%–90%) 3 days after thawing; therefore, the cells need to be passed 3 days after thawing. Otherwise, some clusters of cells are detached (see day 6 in Figure 1). Thus, our protocol established IMhu to be passed 3 days after thawing, and about twice weekly after the first passage. In our experience, cells can be used for experiments up to 10 passages.

**Figure 2:**
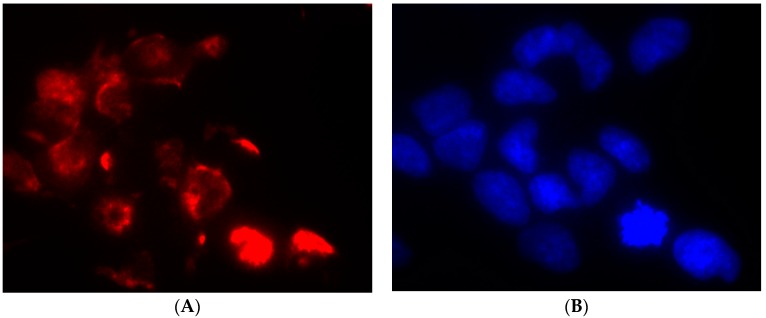
CD11b staining in IMhu. Cells were stained with antibodies against the CD11b surface antigen. (**A**) Panel A shows positive staining for CD11b and (**B**) panel B shows nuclear DAPI staining. Magnitude 40×.

IMhu are marketed as human microglia cells; in order to validate this datum, we carried out an immunofluorescence staining for a marker specific of human microglia-macrophage lineage, namely CD11b. One hundred percent cells were found positive for CD11b (Figure 2).

**Figure 3:**
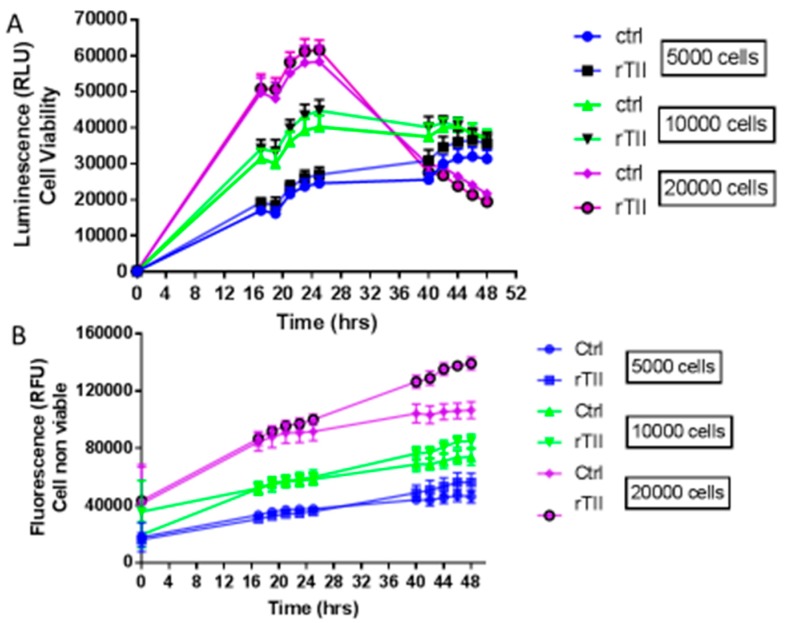
Evaluation of cell viability and cell mortality of IMhu Cells. Panel (**A**) shows the growth of IMhu cell line under basal conditions in plain growth media and in the presence of TII with different number of seeded cells. Panel (**B**) shows cell mortality, respectively, in the presence of the indicated treatment. The fluorescence data indicates the non-viable cells, whereas the luminescence data indicates the viable cells. Two-way ANOVA analysis was carried out.

In order to characterize cell growth and lethality, real time course experiments (0–48 h) were carried out seeding 5000, 10,000 or 20,000 cells per well, and measuring vitality and cellular mortality in real-time (Figure 3). In these experiments, two different groups of microglial cells were investigated: a control group treated with 1% serum medium, and a group stimulated with a mix of pro-inflammatory cytokines, TNFα, IL1β, and IFNϒ, referred to as TII group. TII stimulus is used to activate IMhu toward a M1 phenotype. Figure 3 shows that cells, regardless of density at baseline, reach the highest number at 24 h of treatments (that means 48 h after seeding). This finding suggests setting up experiments with 24-h treatments. As far as lethality is concerned, the number of dead cells increases significantly after 24 h of treatment, especially in the group seeded at a density of 20,000 cells/well. In no case, TII proved to affect cell viability (Figure 3).

**Figure 4:**
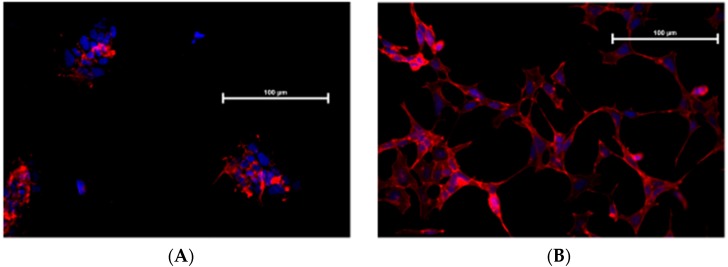
F-actin immunostaining of IMhu. Morphological modifications of IMhu cells, observed 24 h post treatments, with a staining of F-actin with phalloidin-TRIC and fluorescence microscopy. (**A**) Cells under basal conditions or (**B**) after TII stimulation for 24 h. Magnitude 20×.

Figure 4 shows that TII exposure modifies IMhu cell morphology. In the control group, phalloidin staining shows that the cells maintain a triangular shape with few branches, and are grouped in islets whereas, in the group treated with TII, no grouping in islet is observed, but single cells form a network with a large number of ramifications, which is the morphological presentation of activated microglia 

**Figure 5:**
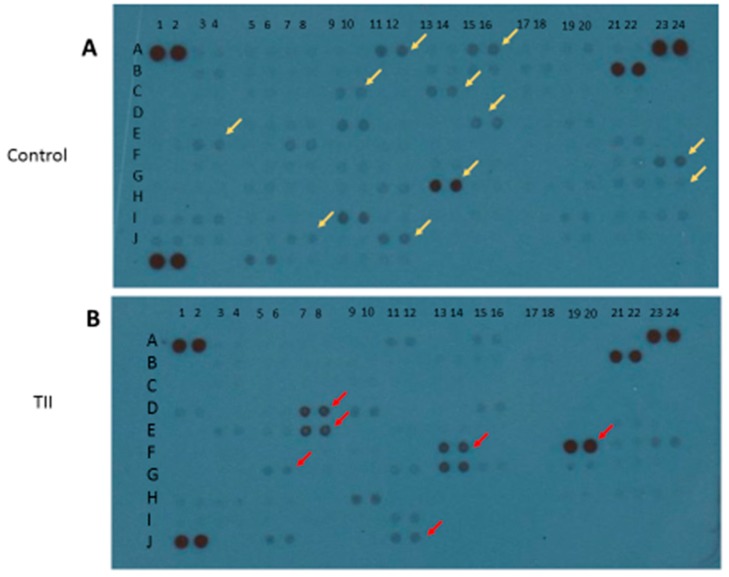
Qualitative analysis of multiple cytokines, chemokines, growth factors, and other soluble proteins in cellular lysates of IMhu after TII stimulation. Panel (**A**) shows the results from control group, and panel (**B**) shows the results from TII group. Yellow arrows indicate the down-regulated factors; red arrows indicate the up-regulated factors.

To characterize the activation profile of TII-stimulated IMhu cells, we used the Cytokine XL kit from R & D (Minneapolis, MN, USA), a membrane-based antibody array for the parallel determination of selected human cytokines and chemokines. After 24 h of stimulation with TII, cell lysates were examined: 17 factors out of a total of 100 cytokines and chemokines were modified. In particular, 6 out of 17 factors were up-regulated, namely IFN-ϒ, IL32, IFN-ϒ-inducible protein 10 (IP-10), Lipocalin-2, the chemokine IL8, and the adhesion molecule vascular cell adhesion molecule 1 (Vcam-1). Conversely, 11 out of 17 factors were down-regulated, in particular, angiopoietin-2, brain-derived neurotrophic factor (BDNF), basic fibroblast growth factor (FGF basic), FGF-19, IL1α, IL5, Kallikrein 3, migration inhibitory factor (MIF), MMP-9, thymus- and activation-regulated chemokine TARC/CCL17 and transferrin receptor protein 1 TFR/CD71 (Figure 5 and Table 1).

**Figure 6:**
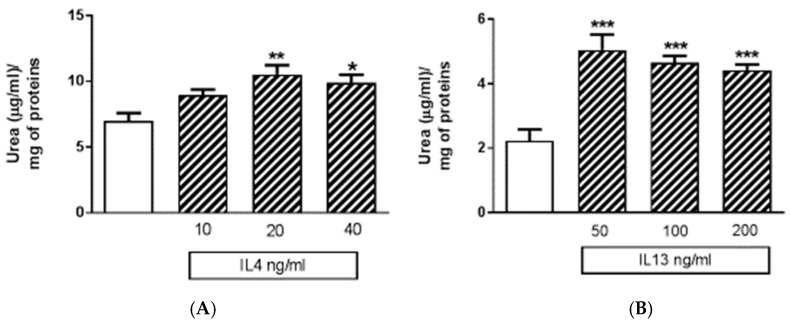
Effects of the human recombinant interleukin 4 (IL4) and human recombinant interleukin 13 (IL13) on urea release. (**A**) The urea content in IMhu cells, under basal condition (white) and after IL4 challenge (striped columns). (**B**) The urea content in IMhu cells, under basal condition (white) and after IL13 challenge (striped columns). The data are obtained after 24 h of incubation. * *p* < 0.05, ** *p* < 0.01, *** *p* < 0.0001 versus control. One-way ANOVA analysis was carried out.

**Figure 7:**
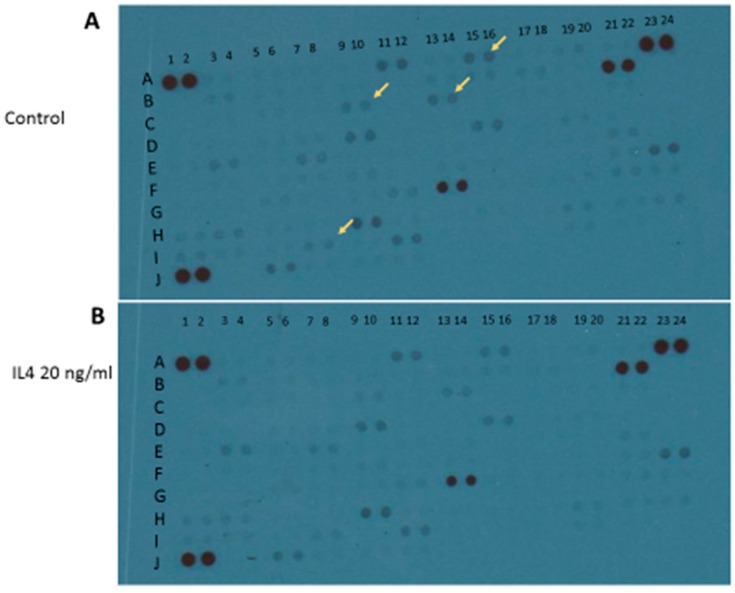
Qualitative analysis of multiple cytokines, chemokines, growth factors, and other soluble proteins in cellular lysates of IMhu after IL4 stimulation. Panel (**A**) shows the results from control group and panel (**B**) shows the results from IL4 group. Yellow arrows indicate the down-regulated factors.

Apart from M1 activation, a series of experiments was carried out with IL4 or IL13 in order to evaluate M2 activation. The release of urea was taken as a M2 marker in preliminary 24-h experiments (Figure 6). Both IL4 and IL13 were able to significantly increase urea levels; statistically significant increases were obtained with IL13 in the range of concentrations 50–100–200 ng/mL, and with IL4 in the range of concentrations 10–20–40 ng/mL (Figure 6). At the end of experiments, cell viability was assessed by measurement of the activity of LDH released in the incubation media. None of the treatments performed significantly increased LDH activity above control levels; in particular, control has been taken as 100% and the data were expressed as % of control: 40 ng/mL IL4 measured 67% ± 3.9% and 200 ng/mL IL13 93% ± 8.2%. Responses to IL13 stimulation were dependent on the medium used, since significant increases in urea levels were observed when IL13 was dissolved in DMEM 1% serum; whereas, no increase was found if the medium was DMEM/F12 (data not shown). Because of such variability, in all subsequent experiments IL4 20 ng/mL was preferred as a tool for M2 phenotype induction. Using IL4 as a stimulus, 4 out of 100 factors assessed with the above-mentioned cytokine XL kit were modified. In particular, BDNF, FGF basic, FGF-19, and TARC/CC17 factors were down regulated (Figure 7 and Table 2).

**Figure 8:**
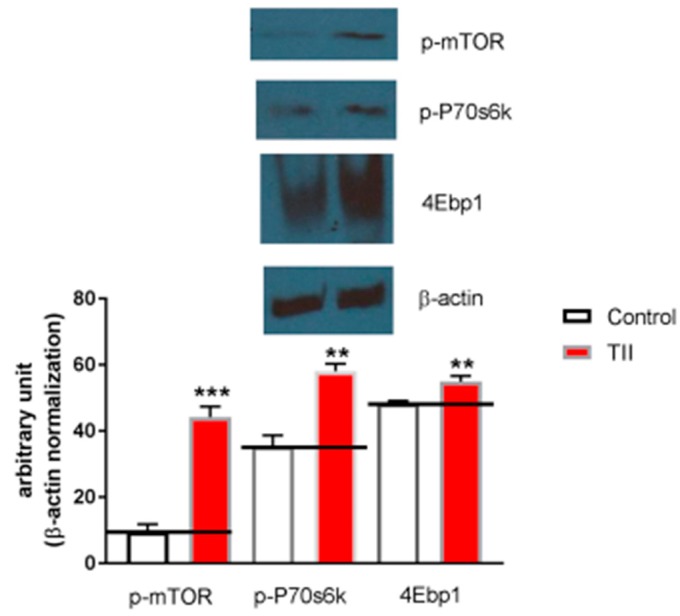
Analysis of mTOR phosphorylation during IMhu activation. (**A**) Whole-cell lysates were prepared from IMhu incubated for 2 h as indicated. Equal amounts of proteins were analyzed by western blot for phosphorylated mTOR kinase (p-mTOR), phosphorylated P-70S6K, and 4EBP1. The same blots were subsequently probed for β-actin, lower gel. (**B**) Quantitation of densitometry where p-mTOR, p-p70S6K, and 4EBP1 values are reflected relative to those for β-actin. Data are expressed as means ± SEM of *n* = 1 replicates for each group, each assayed in triplicates. Representative of two different experiments. Data were analyzed by one-way ANOVA followed by Bonferroni’s post hoc test. ** *P* < 0.01 and *** *P* < 0.0001 versus control.

Finally, in order to ascertain whether IMhu microglia cell line is a useful model to investigate mTOR pathway, 2-h experiments were conducted in the presence of TII analyzing ser2448—taken as parameter of mTOR activation. We found that phosphorylated-mTOR is significantly upregulated by TII administration, along with target proteins downstream of mTOR, p70S6K, and 4EBP1 (Figure 8).

## 4. Discussion

In this work, we carried out an extensive characterization of IMhu immortalized microglia cell line, involving the functional response of IMhu to a large variety of stimuli. In particular, we investigated morphology, mortality, and changes in the production of different cytokines and chemokines, both under basal conditions and after stimulation. Moreover, western blotting analysis was conducted on phospho-mTOR (Ser 2448) and downstream parameters, p-P70S6K and 4EBP1, in order to understand if IMhu can be used for evaluations of mTOR pathway.

Firstly, an evaluation of microglia-macrophage cell marker was conducted in order to confirm that IMhu are indeed belonging to the microglia cell lineage (Figure 2). A number of markers are normally used to address this issue, namely CD68, CD11b, or Iba-1 [16]. Among these markers, we focused on CD11b, a member of the integrin family that couples with CD18 to form CR3 heterodimers. CD11b is expressed on the surface of many leukocytes including monocytes, neutrophils, natural killer cells, granulocytes, and macrophages/microglia. CD11b regulates leukocyte adhesion and migration. Apart from its role in adhesion, CD11b acts as a receptor for complement C3bi, mediating complement-coated particle uptake. Studies on CD11b antibodies lead to identify CD11b as a receptor for fibrinogen gamma chain, factor X, and ICAM1, with a possible involvement in cell-mediated cytotoxicity, chemotaxis, and phagocytosis [17]. A modified expression of Cd11b is observed in different states of microglia cell activation; these changes were out of scope of investigation in this work.

In the study, we showed that exposure to TII is not associated to toxicity (Figure 3); these findings confirm previous observations reported by Reiner et al. [15], obtained using TII in the same range of doses. We gained initial evidence that TII challenge is able to induce changes in IMHu phenotype with morphology experiments (Figure 4). These findings are in agreement with previous observations by ourselves and other groups, obtained with different microglia sources and stimuli [18,19,20]. In all cases, microglia are displayed as single cells forming a network, rather than a cluster of cells, and a large number of ramifications.

In parallel with morphological changes, a pro-inflammatory activation leading to M1 phenotype is also demonstrated by the up-regulation of 6 out of 17 modified inflammatory mediators, in particular IFN-ϒ, IL32, IP-10, lipocalin-2, and the chemokine IL8 and Vcam-1 (Figure 5). In general, most of the above factors are up-regulated in the presence of IFN, and exacerbate inflammation. Conversely, 11 out of the modified 17 factors are down-regulated. These factors, namely angiopoietin-2, BDNF, FGF basic, FGF-19, IL1α, IL5, kallikrein 3, MIF, MMP-9, TARC/CCL17, and TFR/CD71 (Figure 5), are growth factors involved mainly in cell migration.

We also showed that exposures to TII as short as 2 h were associated with significant changes in the entire mTOR pathway (Figure 8); this finding is in keeping with previous observations by ourselves and other groups in other microglia models [21,22,23].

As far as microglial activation towards an M2 phenotype is concerned, the cytokines IL4 or IL13 alone or in association with IL10 are typically used as stimuli. In our experimental paradigm, we first chose to try cytokines alone, but we did not obtain reproducible data, so we decided not to go ahead with the stimulation in association. In fact, we failed to activate IMHU towards a complete type M2 phenotype: while we were able to induce increases in urea release, such increase was not fully reproducible. Moreover, stimulation with IL4 elicited an increase production of factors that are not taken as markers of M2 profiling. In addition, the data obtained after activation with IL4 or IL13 depend on the medium used, and, therefore, this type of activation deserves a detailed study to find the right conditions. However, in our opinion too strict and complicated protocol lead the use of a cellular line too far from the possibility of having translational data.

## 5. Conclusions

In conclusion, IMhu cells proved to be a useful experimental model to investigate the physiopathology of microglia cells, including pathological conditions that involve the mTOR pathway. IMhu cells partially addressed a need for human models of microglia; however, considering that in vitro studies in the field usually require demonstrations based on more than one cellular model, the quest for additional tools remains still unsatisfied.

## Figures and Tables

**Table 1:** Panel of cytokines, chemokines, growth factors, and other soluble proteins modified by TII stimulation.

A ^†^	B ^‡^	hTII ^$^
A11–12	Angiopoietin-2	−
A15–16	BDNF	−
C9–10	FGF basic	−
C13–14	FGF-19	−
D7–8	IFN-y	+
D15–16	IL-1α	−
E3–4	IL-5	−
E7–8	IL-8	+
F13–14	IL-32α/β/γ	+
F19–20	IP-10	+
F23–24	Kallikrein 3	−
G5–6	Lipocalin-2	+
G13–14	MIF	−
G23–24	MMP-9	−
I7–8	TARC/CCL17	−
I11–12	TfR/CD71	−
J11–12	VCAM-1	+

**^†^**, indicates the position on membrane of Figure 7; **^‡^**, indicates the name of modified factors; **^$^**, indicates the results under IL4 stimulation: “−“ indicates that the factor is down-regulated.

**Table 2:** Panel of cytokines, chemokines, growth factors, and other soluble proteins modified by IL4 stimulation.

A ^†^	B ^‡^	hIL4 ^$^
A15–16	BDNF	−
C9–10	FGF basic	−
C13–14	FGF-19	−
I7–8	TARC/CCL17	−

**^†^**, indicates the position on membrane of Figure 7; **^‡^**, indicates the name of modified factors; **^$^**, indicates the results under IL4 stimulation: “−“ indicates that the factor is down-regulated.
